# Radiobiological evaluation considering setup error on single‐isocenter irradiation in stereotactic radiosurgery

**DOI:** 10.1002/acm2.13322

**Published:** 2021-06-20

**Authors:** Hisashi Nakano, Satoshi Tanabe, Ryuta Sasamoto, Takeshi Takizawa, Satoru Utsunomiya, Madoka Sakai, Toshimichi Nakano, Atsushi Ohta, Motoki Kaidu, Hiroyuki Ishikawa

**Affiliations:** ^1^ Department of Radiation Oncology Niigata University Medical and Dental Hospital Niigata Japan; ^2^ Department of Radiological Technology Niigata University Graduate School of Health Sciences Niigata Japan; ^3^ Department of Radiation Oncology Niigata Neurosurgical Hospital Niigata Japan; ^4^ Department of Radiology and Radiation Oncology Niigata University Graduate School of Medical and Dental Sciences Niigata Japan

**Keywords:** brain metastases, setup error, single‐isocenter technique, stereotactic radiosurgery, tumor control probability

## Abstract

**Purpose:**

We calculated the dosimetric indices and estimated the tumor control probability (TCP) considering six degree‐of‐freedom (6DoF) patient setup errors in stereotactic radiosurgery (SRS) using a single‐isocenter technique.

**Methods:**

We used simulated spherical gross tumor volumes (GTVs) with diameters of 1.0 cm (GTV 1), 2.0 cm (GTV 2), and 3.0 cm (GTV 3), and the distance (*d*) between the target center and isocenter was set to 0, 5, and 10 cm. We created the dose distribution by convolving the blur component to uniform dose distribution. The prescription dose was 20 Gy and the dose distribution was adjusted so that D95 (%) of each GTV was covered by 100% of the prescribed dose. The GTV was simultaneously rotated within 0°–1.0° (*δR*) around the x‐, y‐, and z‐axes and then translated within 0–1.0 mm (*δT*) in the x‐, y‐, and z‐axis directions. D95, conformity index (CI), and conformation number (CN) were evaluated by varying the distance from the isocenter. The TCP was estimated by translating the calculated dose distribution into a biological response. In addition, we derived the x‐y‐z coordinates with the smallest TCP reduction rate that minimize the sum of squares of the residuals as the optimal isocenter coordinates using the relationship between 6DoF setup error, distance from isocenter, and GTV size.

**Results:**

D95, CI, and CN were decreased with increasing isocenter distance, decreasing GTV size, and increasing setup error. TCP of GTVs without 6DoF setup error was estimated to be 77.0%. TCP were 25.8% (GTV 1), 35.0% (GTV 2), and 53.0% (GTV 3) with (*d, δT_,_* *δR*) = (10 cm, 1.0 mm, 1.0°). The TCP was 52.3% (GTV 1), 54.9% (GTV 2), and 66.1% (GTV 3) with (*d, δT_,_* *δR*) = (10 cm, 1.0 mm, 1.0°) at the optimal isocenter position.

**Conclusion:**

The TCP in SRS for multiple brain metastases with a single‐isocenter technique may decrease with increasing isocenter distance and decreasing GTV size when the 6DoF setup errors are exceeded (1.0 mm, 1.0°). Additionally, it might be possible to better maintain TCP for GTVs with 6DoF setup errors by using the optimal isocenter position.

## 1 INTRODUCTION

A single‐isocenter irradiation technique for stereotactic radiosurgery (SRS) and stereotactic radiotherapy (SRT) was previously introduced for multiple brain metastases.^1^, ^2^, ^3^ The advantage of the single‐isocenter technique is that it reduces the dose‐delivery time in comparison with that in conventional multiple‐isocenter irradiation because multiple targets are irradiated simultaneously at a single isocenter.^4^, ^5^ Therefore, the single‐isocenter technique is effective for the irradiation of multiple targets such as brain metastases. However, there is a problem in that the effect of the patient setup error, including translational and rotational errors, is considered to be larger in the single‐isocenter technique than in conventional multiple‐isocenter irradiation. Consequently, the planning isocenter has not been located at the centre of the target in many single‐isocenter cases.^4^, ^5^

The effect of setup error in the single‐isocenter technique has been evaluated in several studies and has been found to depend on the distance from the isocenter and the target size.^6^, ^7^ Chang et al. calculated the CTV margin when the distance from the isocenter and target size were varied to satisfy the target dose coverage using a mathematical model.^6^, ^7^ However, the dose volume for the target with setup error could not be evaluated. Although several researchers have used clinical data to evaluate the dose coverage of targets with setup errors, the relationship between the dose coverage and setup could not be generalized because it was evaluated using clinical data with different parameters such as target size and distance from the isocenter.^8^, ^9^, ^10^ We evaluated the effect of six degrees of freedom (6DoF) setup errors in patient setups on SRS using a single‐isocenter technique.^11^, ^12^ However, the study was a geometrical evaluation, and the penumbra of dose distribution change was not considered. Thus, it is necessary to perform generalization using a mathematical model to clarify the relationship between the target volume dose and setup error in a phantom study when the distance from the isocenter and target size are varied in the single‐isocenter technique. Hence, we calculated the dose volume histograms (DVHs) of targets considering various setup errors, distances from the isocenter, and target sizes using a mathematical model. Moreover, to our knowledge, the optimal isocenter positions were not revealed in previous studies when multiple targets were irradiated with the use of single‐isocenter irradiation. Therefore, the optimal isocenter positions for GTV were calculated by using the relationship between 6DoF setup error, distance from isocenter, and target size.

The tumor control probability (TCP), which is the biologic response obtained using the dose volume information for a target,^13^, ^14^, ^15^, ^16^ provides an effective means of comparing DVHs to evaluate whether the degree of volume dose for the target is optimal. The parameters used to estimate the TCP have been reported based on clinical outcomes for brain metastases using SRS and SRT.^17^, ^18^ In this study, we estimated the TCP by translating the calculated DVHs into biologic responses and TCP with 6DoF setup error at the optimal isocenter position was calculated simultaneously in single‐isocenter irradiation.

## 2 MATERIALS AND METHODS

### 2.A Phantom design and creation of dose distributions

The diameters of the target spheres that served as the simulated gross tumor volumes (GTVs) were set as follows: 1.0 cm (GTV 1), 2.0 cm (GTV 2), and 3.0 cm (GTV 3), and MATLAB ver. 2020a software (MathWorks, Natick, MA, USA) was used. The coordinates (unit: cm) of the GTVs were set such that the distance between the centre of the GTV and the isocenter was 0, 5, and 10 cm.^6^, ^7^, ^19^ The isocenter was set as the origin of the coordinate axes.

**Fig. 1:**
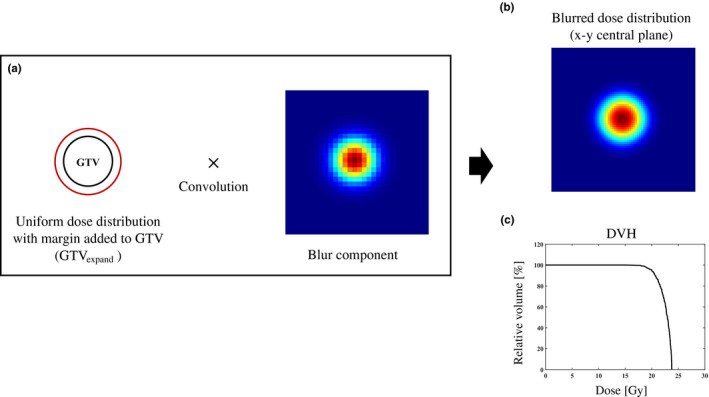
A uniform dose distribution was created by adding a margin (1 mm) to GTV (GTV_expand_) (a). Calculating the dose distribution by blur component of dose following a normal distribution that three‐dimensional normal distribution probability density convolved on creating a uniform dose distribution (b). DVH of GTV that D95(%) of each GTV was covered by 100% of the prescribed dose (20 Gy) (c)

First, a uniform dose distribution was created by adding a margin (1 mm in this study) to each GTV (GTV_expand_) (Fig. 1(a)). Second, the convolution integral was performed on the blur component of the dose to creating a uniform dose distribution, which had a probability density following a three‐dimensional normal distribution, to calculate the blurred dose distribution for each GTV in Equation (1), (2) (Fig. 1(b)). Third, the prescription dose was 20 Gy for each GTV in Equation (3).^18^, ^19^, ^20^, ^21^ The dose distribution was adjusted so that D95 (%) of each GTV was covered by 100% of the prescribed dose (Fig. 1(c)). The maximum GTV dose was set to be approximately 120% (24 Gy) of the prescribed SRS dose for clinical brain metastases (Fig. 1(c)).^18^

$$Blur_{x}=\frac{1}{\sqrt{2\pi }\sigma_{x}}e_{2S\sigma_{x}}^{‐{\left(x‐x^{\prime }\right)}^{2}}$$

$$Blur_{y}=\frac{1}{\sqrt{2\pi }\sigma_{y}}e_{2S\sigma_{y}}^{‐{\left(y‐y^{\prime }\right)}^{2}}$$

(1) $$Blur_{z}=\frac{1}{\sqrt{2\pi }\sigma_{z}}e_{2S\sigma_{z}}^{‐{\left(z‐z^{\prime }\right)}^{2}}$$

(2) $$Blur_{3D}=\;{\mathrm{Blur}}_{x}{\mathrm{Blur}}_{y}{\mathrm{Blur}}_{z}$$

(3) $$Dose_{3D}\left(x,y,z\right)=D_{\mathrm{prescription}}\int \int \int GTV_{\mathrm{expand}}\left(x^{\prime },y^{\prime },z^{\prime }\right)Blur_{3D}dx^{\prime }dy^{\prime }dz^{\prime }$$

### 2.B Calculation of dose coverage reduction with six degree‐of‐freedom setup errors

The calculation model for the translational and rotational errors of a GTV that rotates around the axis passing through the isocenter. The spherical coordinates of GTV are the Cartesian coordinates, that is, x, y, and z. Equation (4) shows the conversion of polar coordinates to the Cartesian coordinate system.

x=dcosφcosθ

y=dcosφsinθ

(4) z=dsinφ

First, the (x, y, z) is rotated around the x‐axis by an angle α, around the y‐axis by an angle β, and around the z‐axis by an angle γ to obtain the (x_δR_, y_δR_, z_δR_) in Eq. (5). The rotational angles of α, β, and γ were the same, and this value was defined as *δR* (δ_rot_ = α, β, γ) in this study.

(5) $$\left(\begin{array}{c} x_{\delta R}\\ y_{\delta R}\\ z_{\delta R} \end{array}\right)=\left(\begin{array}{ccc} \cos\gamma & \sin\gamma & 0\\ ‐\sin\gamma & \cos\gamma & 0\\ 0 & 0 & 1 \end{array}\right)\left(\begin{array}{ccc} \cos\beta & 0 & ‐\sin\beta \\ 0 & 1 & 0\\ \sin\beta & 0 & \cos\beta \end{array}\right)\left(\begin{array}{ccc} 1 & 0 & 0\\ 0 & \cos\alpha & \sin\alpha \\ 0 & ‐\sin\alpha & \cos\alpha \end{array}\right)\left(\begin{array}{c} x\\ y\\ z \end{array}\right)$$

The target position coordinates with the isocenter as a start point were simultaneously rotated clockwise around the x‐, y‐, and z‐axes with *δR* ranging from 0° to 1.0°.

Next, the 6DoF setup error was evaluated by adding a translational error to the (x_δR_, y_δR_, z_δR_). The (x_6DoF_, y_6DoF_, z_6DoF_) was calculated as the translational error *δT* in the positive direction of the x‐, y‐, and z‐axes added to the (x_δR_, y_δR_, z_δR_) (Eq. 6). The translational error *δT* component values were 0.5 mm and 1.0 mm.

(6) $$\left(\begin{array}{c} x_{6DoF}\\ y_{6DoF}\\ z_{6DoF} \end{array}\right)=\left(\begin{array}{c} x_{\delta R}+{\delta T}_{x}\\ y_{\delta R}+{\delta T}_{y}\\ z_{\delta R}+{\delta T}_{z} \end{array}\right)$$

**Fig. 2:**
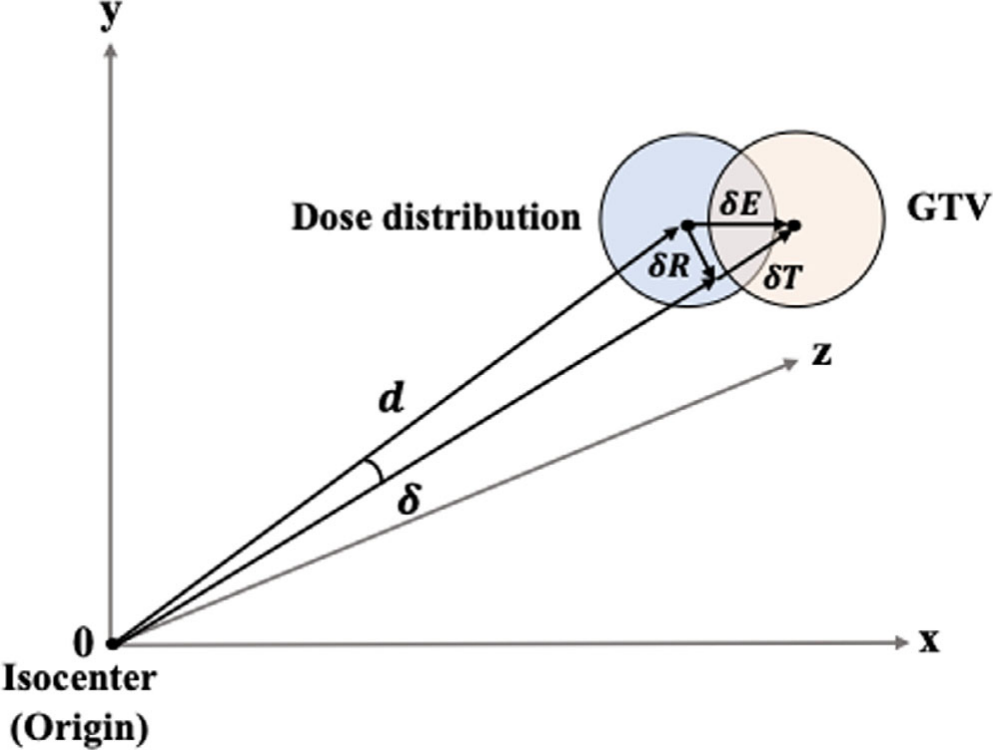
The mathematical calculation model of the 6DoF setup error (δE) was calculated as the sum translational (δT) and rotational (δR) errors for GTV that rotates around the axis passing through the isocenter

The 6DoF setup error δE was calculated as the sum δT+δR, which is the combination of the translational and rotational errors in Figure 2.

The dose coverage reduction for the GTV at each 6DoF setup error was calculated and compared with the case of the setup error at 0°.

### 2.C Evaluation of dosimetric indices with 6DoF setup error

D95 (%) was evaluated for each GTV with a 6DoF setup error while varying the distance from the GTV to the isocenter. The calculated dose distribution was used to evaluate the conformity of the treatment plans for each GTV. The Radiation Therapy Oncology Group (RTOG) conformity index (CI)^22^ and conformation number (CN)^23^ were calculated for each GTV with each plan (Eq. 7, 8).

(7) $$CI=\frac{V_{\mathrm{RX}}}{V_{\mathrm{GTV}}}$$

(8) $$CN=\frac{{\mathrm{TV}}_{\mathrm{RI}}}{\mathrm{TV}}\times \frac{{\mathrm{TV}}_{\mathrm{RI}}}{V_{\mathrm{RI}}}$$

where *V_Rx_* is the volume of the prescription dose and *V_GTV_* is the volume of the GTV. The CI could be evaluated for each target, whether the target volume was over‐ or under‐covered by the prescription volume. The CI of a perfectly conformal plan would be 1, whereas it would be <1 or >1 for a less conformal plan. The CN is the product of a tumor coverage factor and a normal tissue over dosage factor. The *TV_RI_* is target volume covered by the prescription isodose, *TV* is the target volume, and *V_RI_* is volume of the prescription isodose.

### 2.D Estimation of TCP by varying 6DoF setup error

The DVHs with 6DoF setup errors were converted into generalized equivalent uniform doses (gEUDs) using the in‐house software to calculate the biological impact. gEUD is given by Eq. 9:^22^, ^23^

(9) $$gEUD=\;{\left(\sum_{i}^{N}V_{i}D_{i}^{a}\right)}^{\frac{1}{a}}$$

Here, *N* is the number of voxels in the volume of interest and *V_i_* and *D_i_* are the fractional volume and the dose at that volume, respectively. A tumor specific value of *a* = −5 was used in this study.^24^

The biological effectiveness dose (BED) was calculated using a linear–quadratic–cubic model that could fit well the surviving fraction at high dose by Eq. 10 and 11:^25^

(10) $$BED=\;nd\left[1+d/\left(\alpha /\beta \right)‐d^{2}/\left(\alpha /\gamma \right)\right]$$

(11) $$\gamma =\;\frac{\beta }{3D_{1}}$$

Here, *n* is the number of dose fractions and *d* is the dose per fraction. α/β values of 10–15 Gy have been used for tumors,^26^ and α/β = 12 Gy was reflected in the surviving fraction of brain metastases.^16^ *D_1_* is the dose at which the survival curve straightens and was set to 18 Gy^17^ for brain metastases to calculate the coefficient γ. We calculated BED_12_ in this study by converting Eq. 12 using values such as those of α/β and gEUD:^17^, ^27^

(12) $${\mathrm{BED}}_{12}=20\left[1+\left(gEUD/12\right)‐{\left(gEUD\right)}^{2}/648\right]$$

The TCP was evaluated to translate the calculated DVHs into estimated biologic responses. The TCP was estimated by calculating gEUD and BED_12_ using in‐house software (MATLAB) and applying the equation.

(13) $$TCP=\;\frac{{\mathrm{TCP}}_{\max}}{1+{\left(\frac{D_{50}}{{\mathrm{BED}}_{12}}\right)}^{4\gamma_{50}}}$$

TCP_max_, which is the parameter used to calculate the TCP, is the asymptotic local control rate for large *D*. *D*_50_ is the dose corresponding to TCP = 50% with BED_12_, and $\gamma_{50}$ is the slope of the response dose curve. The parameters used to calculate the TCP were TCP_max_ = 86.86%, *D*_50_ = 28.97 Gy, and $\gamma_{50}$ = 1.41 in this study (Eq. 13).^18^ In addition, the maximum and minimum value of 95% confidence interval for the TCP_max_, *D*_50_ and $\gamma_{50}$ were used to assess how much it affected the TCP. The parameters with maximum of 95% confidence for TCP were TCP_max_ were 103.10%, *D*_50_ were 24.80 Gy, and $\gamma_{50}$ was 0.40 (TCP_upper95%conf)_.^16^ Those with minimum of 95% confidence for TCP were 70.62%, 33.14 Gy, and 2.87 (TCP _lower95%conf)_).

### 2.E Calculation of TCP at the optimal isocenter position by using the relationship between 6DoF setup error, distance from isocenter, and GTV size

We set the following coordinates x‐y‐z coordinates of GTV 1, GTV 2, and GTV 3 in condition 1: (5 cm, 0 cm, 0 cm), (0 cm, 5 cm, 0 cm), and (0 cm, 0 cm, 5 cm), respectively. The corresponding coordinates in condition 2 were set at: (10 cm, 0 cm, 0 cm), (0 cm, 10 cm, 0 cm), and (0 cm, 0 cm, 10 cm), respectively. Approximate formula was obtained from the relationship between 6DoF setup error, distance from isocenter, and GTV size for TCP. The coordinates with the smallest TCP reduction rate were calculated by using the approximation formula. The approximation formula with varying distance from isocenter was derived for each GTV with 6DoF setup errors using the results of Figure 4 (Eq. 14).

$$y_{GTV1(0.5\;mm,\;\;0.5^{\circ })}=‐0.09x^{2}+75.80$$

$$y_{GTV1(1.0\;mm,\;\;1.0^{\circ })}=‐0.11x^{2}+5.42x+69.10$$

$$y_{GTV2(0.5\;mm,\;\;0.5^{\circ })}=‐0.05x^{2}+0.11x+76.50$$

$$y_{GTV2(1.0\;mm,\;\;1.0^{\circ })}=‐0.32x^{2}‐0.43x+72.20$$

$${\;y}_{GTV3(0.5\;mm,\;\;0.5^{\circ })}=‐0.03x^{2}+0.07x+77.00$$

(14) $$y_{GTV3(1.0\;mm,\;\;1.0^{\circ })}=‐0.28x^{2}+0.64x+74.1$$

The coordinates of each GTV center and the optimal isocenter were set as (x_GTV_, y_GTV_, z_GTV_) and (x, y, z). The x‐y‐z coordinates that minimize the sum of residual squares (res) that can be obtained from Eq. (14) are the position coordinates of the isocenter that has the lowest rate of reduction in TCP of all three GTVs. We minimized the residual sum of squares at 6DoF setup error by solving an optimization problem, and the position x‐y‐z coordinates of the optimal isocenter were calculated using MATLAB (Eq. 15).

$$res1=\sqrt{{\left(x‐x_{{\mathrm{GTV}}_{1}}\right)}^{2}+{\left(y‐y_{{\mathrm{GTV}}_{1}}\right)}^{2}+{\left(z‐z_{{\mathrm{GTV}}_{1}}\right)}^{2}}$$

$$res2=\sqrt{{\left(x‐x_{{\mathrm{GTV}}_{2}}\right)}^{2}+{\left(y‐y_{{\mathrm{GTV}}_{2}}\right)}^{2}+{\left(z‐z_{{\mathrm{GTV}}_{2}}\right)}^{2}}$$

(15) $$res3=\sqrt{{\left(x‐x_{{\mathrm{GTV}}_{3}}\right)}^{2}+{\left(y‐y_{{\mathrm{GTV}}_{3}}\right)}^{2}+{\left(z‐z_{{\mathrm{GTV}}_{3}}\right)}^{2}}$$

The center points of GTV1, 2, and 3 were defined as P_GTV1_, P_GTV2_, and P_GTV3_, and the calculated point is defined as P_(x,y,z)_. The optimization was performed using the constraints shown in Eqs. (16) – (20) so that the calculated optimal isocenter coordinates remains between GTV1,2, and 3 when the optimal isocenter coordinates were calculated (in which s and t are coefficients). The optimal isocenter coordinate position is defined as the point at which the total of the TCP reduction rates of the three GTVs is minimized. The optimal isocenter position was derived for the GTV positions under conditions 1 and 2. We calculated the TCP for each GTV and the total TCP reduction for all GTVs using the derived optimal isocenter.

(16) $$\min_{x,y,z}\left({res1}^{2}+{res2}^{2}+{res3}^{2}\right)$$

(17) $$\vec{P_{GTV1}P_{\left(x,y,z\right)}}=s\vec{P_{GTV1}P_{GTV2}}+t\vec{P_{GTV1}P_{GTV3}}$$

(18) s>0

(19) t>0

(20) s+t>1

We also calculated the decreasing dose coverage when the isocenter position was placed at the center of gravity or the center of the three GTVs, in order to compare these values with the rate of the decrease in dose coverage of each GTV. In conditions 1 and 2, the positions of the center of gravity for the GTVs were (x, y, z) = (0.40 cm, 1.38 cm, 3.22 cm) and (0.83 cm, 2.72 cm, 6.14 cm), and those of the centers of the GTVs were (x, y, z) = (1.67 cm, 1.67 cm, 1.67 cm) and (3.33 cm, 3.33 cm, 3.33 cm), respectively.

## 3 RESULTS

### 3.A Dosimetric indices for each target with the distance from the isocenter and various 6DoF setup errors

**Fig. 3:**
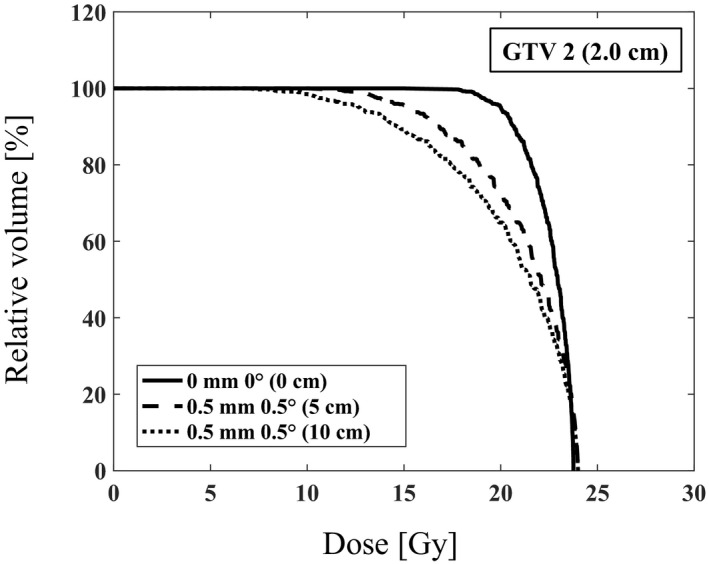
The calculated the dose volume histogram with the 5 and 10 cm distance from the isocenter, (0.5 mm, 0.5°) setup errors for GTV 2 (2.0 cm)

We calculated the DVH for each GTV considering 6DoF setup errors by varying the distance from the isocenter to the GTV. Figure 3 shows the calculated DVHs of GTV 2 (2.0 cm) with 0.5 mm translational error (δT) and 0.5° rotational error (δR) and distances of 5 cm and 10 cm. The dosimetric indices for each GTV were calculated as functions of the distance from the isocenter to the GTV, 6DoF setup error, and GTV diameter (Table 1). As shown in Table 2, D95 (%) is 14.3 Gy for GTV 1 (1.0 cm), 15.2 Gy for GTV 2 (2.0 cm), and 16.5 Gy for GTV 3 (3.0 cm) with (*d*, *δT*, *δR*) = (5 cm, 0.5 mm, 0.5°), respectively. The corresponding values with (*d*, *δT*, *δR*) = (10 cm, 1.0 mm, 1.0°) are 8.2 Gy for GTV 1 (1.0 cm), 8.8 Gy for GTV 2 (2.0 cm), and 9.9 Gy for GTV 3 (3.0 cm), respectively. The CI and CN values with (*d*, *δT*, *δR*) = (5 cm, 0.5 mm, 0.5°) are 0.68 and 0.46 for GTV 1 (1.0 cm), 0.72 and 0.52 for GTV 2 (2.0 cm), and 0.81 and 0.66 for GTV 3 (3.0 cm), respectively. The corresponding values for (*d*, *δT*, *δR*) = (10 cm, 1.0 mm, 1.0°) are 0.48 and 0.23 for GTV 1 (1.0 cm), 0.55 and 0.30 for GTV 2 (2.0 cm), and 0.64 and 0.41 for GTV 3 (3.0 cm), respectively (Table 1).

**Table 1:** The dosimetric indices and TCP for each GTV as a function of the distance from the isocenter to the GTV, the 6 DoF setup error, and the diameter of GTV

GTV (cm)	0	1		2		3	
6 DoF setup error (mm, °)		0.5	1.0	0.5	1.0	0.5	1.0
Dis (cm)	0	5	10	5	10	5	10
D95% (Gy)	20.0	14.3	8.2	15.2	8.8	16.5	9.9
CI	0.95	0.68	0.48	0.72	0.55	0.81	0.64
CN	0.95	0.46	0.23	0.52	0.30	0.66	0.41
gEUD (Gy)	21.8	18.1	3.1	19.4	4.7	21.0	8.0
BED_12_ (Gy)	41.7	40.1	24.9	40.7	27.1	41.4	31.4
TCP (%)	77.0	73.4	25.8	75.8	35.6	76.6	53.0

**Table 2:** The effect of varying calculating parameters to estimate the TCP for each GTV with GTV size, the distance from isocenter, and 6 DoF setup errors

GTV (cm)	0	1		2		3	
6 DoF setup error (mm, °)		0.5	1.0	0.5	1.0	0.5	1.0
Dis (cm)	0	5	10	5	10	5	10
TCP_upper95%conf_ (%)	60.9	58.4	35.8	60.0	43.4	60.6	49.3
TCP_lower95%conf_ (%)	70.4	70.2	39.9	70.4	52.1	70.4	66.2

### 3.B TCP estimation for GTVs with various distances from the isocenter and setup errors

**Fig. 4:**
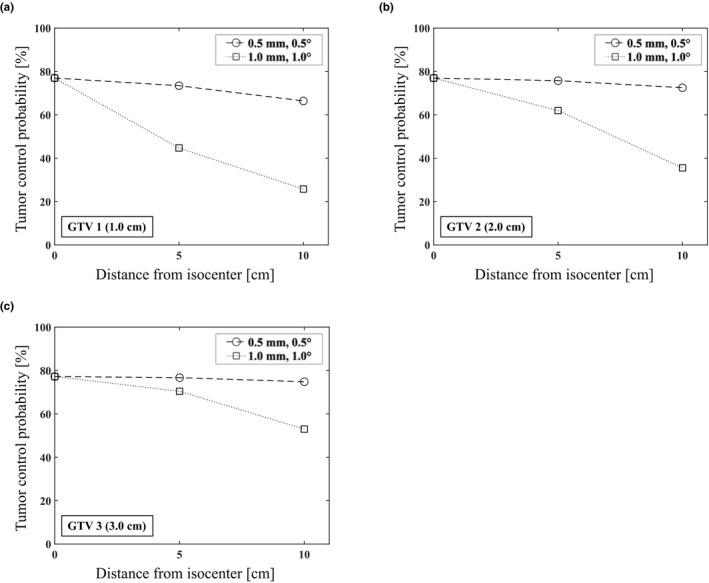
Tumor control probability for GTV 1 (a), GTV 2 (b), and GTV 3 (c) with varying distance and 6DoF setup error

Table 1 showed the parameters such as gEUD and BED_12_ that were used to estimate the TCP for various GTV sizes, setup errors, and distances. The TCP of the GTV without setup error was calculated to be 77.0% using the parameters in this study.^17^ The TCP for each GTV was estimated using various distances from the isocenter and setup errors (Fig. 4). The TCPs for GTV 1 (1.0 cm) were 73.4%, that for GTV 2 (2.0 cm) was 75.8%, and that for GTV 3 (3.0 cm) was 76.6% with (*d*, *δT*, *δR*) = (5 cm, 0.5 mm, 0.5°), respectively (Table 1). The TCPs for GTV 1 (1.0 cm) were 25.8%, that for GTV 2 (2.0 cm) was 35.6%, and that for GTV 3 (3.0 cm) was 53.0% with (*d*, *δT*, *δR*) = (10 cm, 1.0 mm, 1.0°), respectively.

The effect of varying calculating parameters to estimate the TCP for each GTV with GTV size, the distance from isocenter, and 6DoF setup errors (Table 2). The TCP_upper95%conf_ and TCP_lower95%conf_ of the GTV without setup error were calculated to be 60.9% and 70.4%. The TCP_upper95%conf_ for GTV 1 (1.0 cm) was 35.8%, that for GTV 2 (2.0 cm) was 43.4%, and that for GTV 3 (3.0 cm) was 49.3% with (*d*, *δT*, *δR*) = (10 cm, 1.0 mm, 1.0°), respectively (Table 4). The corresponding values of TCP_lower5%conf_ for GTV 1 (1.0 cm) was 39.9%, that for GTV 2 (2.0 cm) was 52.1%, and that for GTV 3 (3.0 cm) was 66.2%, respectively.

### 3.C Calculation of TCP reduction for GTVs at the optimal isocenter positions

**Fig. 5:**
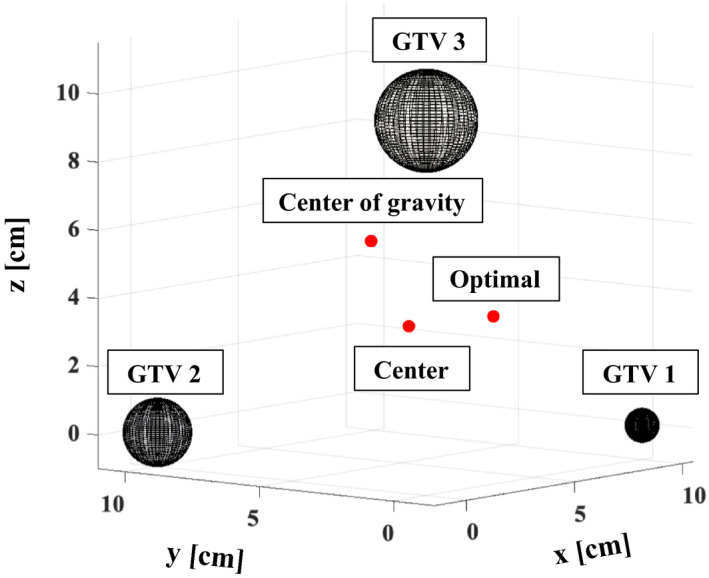
The positions of the isocenter with calculated optimal, center of gravity, and center with GTV 1, GTV 2, and GTV 3 in x‐y‐z coordinates with (*d*, *δT*, *δR*) = (10 cm, 1.0 mm, 1.0°)

Figure 5 shows the positions of the isocenter with calculated optimal, center of gravity, and center with GTV 1, GTV 2, and GTV 3 in x‐y‐z coordinates with (*d*, *δT*, *δR*) = (10 cm, 1.0 mm, 1.0°). The optimal isocenter positions were (x, y, z) = (3.77 cm, 1.54 cm 1.23 cm) and (3.35 cm, 1.67 cm 1.65) with (*d*, *δT*, *δR*) = (5 cm, 0.5 mm, 0.5°) and (5 cm, 1.0 mm, 1.0°) in condition 1. The TCP reduction for GTV 1 was 75.7% and 60.5% (Table 3); those for GTV 2 were 76.3% and 67.2%, and those for GTV 3 were 76.9% and 73.2%. Those of TCP for GTV 1, GTV 2, and GTV 3 were 74.6%, 75.9%, 76.9% with (5 cm, 0.5 mm, 0.5°) when the position of the isocenter was the center of gravity, and they were 50.9%, 63.4%, and 74.4% when the isocenter position was the center of gravity with (5 cm, 1.0 mm, 1.0°). When the isocenter position was the center of the GTVs, those were 74.8%, 76.3%, and 76.9% with (5 cm, 0.5 mm, 0.5°), and 52.3%, 67.2%, and 73.2% with (5 cm, 1.0 mm, 1.0°).

**Table 3:** The TCP (%) and total TCP reduction (%) with the 6DoF setup error when the isocenter is at the calculated optimal position, the center of gravity, and the center of the GTVs in condition 1

6DoF setup error (mm, °)	0.5			1.0		
Isocenter position (x, y, z) (cm)	Optimal (3.77, 1.54, 1.23)	Center of gravity (0.40, 1.38, 3.22)	Center (1.67, 1.67, 1.67)	Optimal (3.35, 1.67, 1.65)	Center of gravity (0.40, 1.38, 3.22)	Center (1.67, 1.67, 1.67)
GTV1: 1.0 cm (%)	75.7	74.6	74.8	60.5	50.9	52.3
GTV2: 2.0 cm (%)	76.3	75.9	76.3	67.2	63.4	67.2
GTV3: 3.0 cm (%)	76.9	76.9	76.9	73.2	74.4	73.2
Total reduction (%)	2.1	3.6	3.0	30.1	42.3	38.3

The calculated optimal isocenter were (x, y, z) = (7.25 cm, 3.18 cm 2.75) and (6.68 cm, 3.33 cm 3.32) with (*d*, *δT*, *δR*) = (10 cm, 0.5 mm, 0.5°) and (10 cm, 1.0 mm, 1.0°) in condition 2. The TCP for GTV 1 were 75.1% and 52.3% (Table 4); those for GTV 2 were 74.9% and 54.9%, and those for GTV 3 were 76.0% and 66.1%. The reduction of TCP at the center of gravity for GTV 1, GTV 2, and GTV 3 were 70.8%, 73.2%, 76.8% with (10 cm, 0.5 mm, 0.5°), and they were 35.4%, 41.1%, and 72.5% with (10 cm, 1.0 mm, 1.0°). When the isocenter position was the center of the GTVs, those were 71.6%, 75.0%, and 76.8% with (10 cm, 0.5 mm, 0.5°), and 37.8%, 54.9%, and 66.1% with (10 cm, 1.0 mm, 1.0°).

**Table 4:** The TCP (%) and total TCP reduction (%) with the 6DoF setup error when the isocenter is at the calculated optimal position, the center of gravity, and the center of the GTVs in condition 2

6DoF setup error (mm, °)	0.5			1.0		
Isocenter position (x, y, z) (cm)	Optimal (7.25, 3.18, 2.75)	Center of gravity (0.83, 2.72, 6.14)	Center (3.33, 3.33, 3.33)	Optimal (6.68, 3.33, 3.32)	Center of gravity (0.83, 2.72, 6.14)	Center (3.33, 3.33, 3.33)
GTV1: 1.0 cm (%)	75.1	70.8	71.6	52.3	35.4	37.8
GTV2: 2.0 cm (%)	74.9	73.2	75.0	54.9	41.1	54.9
GTV3: 3.0 cm (%)	76.0	76.8	76.2	66.1	72.5	66.1
Total reduction (%)	5.0	10.2	8.2	57.7	82.0	72.2

## 4 DISCUSSION

We estimated the TCP by translating the DVHs calculated for the GTVs to evaluate the biologic response considering the setup error with various distances from the isocenter and GTV sizes using mathematical models. The estimated TCP in this study was derived using parameters that were statistically well fitted for 12 months local control rates.^17^ By using a mathematical model, it was possible to evaluate the effects of the GTV size, distance from the isocenter to the target, and setup error without being affected by the data variation that occurs when using clinical data. The TCP of the GTV significantly decreased with increasing distance from the isocenter, decreasing GTV size, and increasing setup error in this study (Fig. 4). Image‐guided radiation therapy (IGRT) imaging systems have been used previously to improve the accuracy of patient localization setup for clinical brain SRS.^28^, ^29^, ^30^ However, these systems are limited in that they can correct the translational and rotational errors with accuracies of only about 0.5 mm and 0.5°, respectively.^29^, ^30^, ^31^, ^32^, ^33^ Therefore, it is difficult to verify the translational and rotational setup errors within 0.5 mm and 0.5°, respectively, which are the clinical limits of the respective patient setup errors. The TCP of GTV 1 was decreased by 73.4% at 5 cm distance and by 66.4% at 10 cm distance compared to its value of 77.0% without setup errors when the limit values of an IGRT system were considered. Similarly, the TCP of GTV 2 was decreased to 75.8% and 72.5% and that of GTV 3 was decreased by 76.6% and 74.8% at distances of 5 cm and 10 cm, respectively (Fig. 4). Wiggenraad et al. concluded that a BED_12_ value of at least 40 Gy was necessary to obtain a 12 months local control rate (TCP) of 70% for brain metastases using single‐fraction SRS.^17^ Considering the 70% TCP value, the limit conditions were (*d*, *δT*, *δR*) = (5 cm, 0.5 mm, 0.5°) for GTV 1 and (10 cm, 0.5 mm, 0.5°) for GTV 2 and GTV 3 in the single‐isocenter technique. When (*δT*, *δR*) = (1.0 mm, 1.0°) was set as the setup error, the 70% TCP for GTVs could not be satisfied except for GTV3 with 5 cm distance in this study.

We calculated the TCP reduction of GTVs at the optimal isocenter position by using the relationship between the distance from the isocenter (Tables 3, 4). The total of TCP reductions of GTVs at the optimal isocenter were 2.1% and 30.1% with (*d*, *δT*, *δR*) = (5 cm, 0.5 mm, 0.5°) and (5 cm, 1.0 mm, 1.0°) (Table 3). On the other hand, the total of TCP reduction at the position that isocenter was center of gravity and center of GTVs were 3.6%, 3.0%, 42.3%, and 38.3%. The calculated optimal isocenter coordinates minimize the total of the reduction rates of the three GTVs compared to the case where the center of gravity or center of GTVs has an isocenter (Tables 3, 4). Stanhope C, et al. evaluated the CI, gradient index, and heterogeneity index as functions of distance from isocenter for GTV with rotational error in single‐isocenter technique when the position of the isocenter was varied.^10^ Comparing the evaluation of Stanhope C, et al. that the dose parameters for large and small targets were a clear tradeoff with the CI value of the model proposed in this study, the results of this study are considered to be valid. Furthermore, they concluded that the isocenter position weighted by target volume should be avoided when the distance between the target and the isocenter was large. It was considered that the derivation of the isocenter position in this study was appropriate since the effect of the isocenter position on the GTV was consistent with the tendency. Therefore, it might be possible to suppress the decreasing of TCP with 6DoF setup errors by using the optimal isocenter coordinates.

Furthermore, we estimated TCP from the resulting DVH by using the proposed mathematical model using the calculation parameters with TCP_max_, *D*_50_, and $\gamma_{50}$ in this study. However, TCP was affected by the calculation parameters such as TCP_max_, *D*_50_, and $\gamma_{50}$. Table 2 showed the effect of varying calculating parameters to estimate the TCP for each GTV with GTV size, the distance from isocenter, and 6DoF setup errors. Therefore, the TCP was affected by these parameters, further studies were necessary to evaluate how the calculation parameters of TCP estimation for GTV. In addition to GTV evaluation, normal brain volume constraints such as V_12Gy_ and brain necrosis is an adverse event of concern in SRS using a single‐isocenter technique.^34^, ^35^ Table 5 showed the robustness of TCP with 6DoF setup error as a function using a couple different target margins (1.0 mm, 2.0 mm) for GTV. The robustness of TCP with 6DoF setup error was improved by adding the margin to GTVs. However, the greater the impact on the surrounding normal brain would be significant when the larger the margin was added to the GTV. It is necessary to incorporate the evaluation of volume constraints and normal tissue complication probability (NTCP) for normal brain into the creating mathematical model and perform further evaluation.

**Table 5:** Evaluating the robustness of TCP with 6DoF setup error as a function using a couple different target margins

Margin (mm)	1.0				2.0			
Distance (cm)	5		10		5		10	
6 DoF setup error (mm, °)	0.5	1.0	0.5	1.0	0.5	1.0	0.5	1.0
GTV1: 1.0 cm (%)	74.0	48.9	67.9	27.1	74.5	52.7	69.3	28.8
GTV2: 2.0 cm (%)	76.1	64.4	73.3	38.4	76.3	66.4	73.9	41.6
GTV3: 3.0 cm (%)	76.6	71.1	74.8	57.4	76.6	71.4	74.7	62.1

This research has two limitations. First, only a single target was evaluated to estimate the TCP, rather than evaluating the TCPs of multiple targets simultaneously. Second, the dose distribution changes with changes in path length and electron density and motion of the immobilization system were not considered.

## 5 CONCLUSIONS

The TCP in SRS for multiple brain metastases may decrease with increasing distance from the isocenter and decreasing GTV size when the 6DoF setup errors are exceeded (1.0 mm, 1.0°) with single‐isocenter technique. Additionally, it might be possible to better maintain TCP for GTVs with 6DoF setup errors by using the optimal isocenter position.

## AUTHOR CONTRIBUTIONS

H. Nakano and R. Sasamoto designed the study and performed the experiments. S. Tanabe, S. Utsunomiya, T. Takizawa, and M. Sakai advised on the content of the study. T. Nakano, A. Ohta, M. Kaidu, and H. Ishikawa supervised and reviewed the manuscript.

## CONFLICT OF INTEREST

The authors have no conflict of interest to declare.

## Data Availability

The data that support the findings of this study are available from the corresponding author upon reasonable request.
